# Medicinal Plants Used as Antitumor Agents in Brazil: An Ethnobotanical Approach

**DOI:** 10.1155/2011/365359

**Published:** 2011-03-08

**Authors:** Joabe Gomes de Melo, Ariane Gaspar Santos, Elba Lúcia Cavalcanti de Amorim, Silene Carneiro do Nascimento, Ulysses Paulino de Albuquerque

**Affiliations:** ^1^Departamento de Biologia, Universidade Federal Rural de Pernambuco, Rua Dom Manoel de Medeiros, s/n, 52171-900 Recife, PE, Brazil; ^2^Departamento de Ciências Farmacêuticas, Centro de Ciências da Saúde, Universidade Federal de Pernambuco, Avenida Prof. Arthur de Sá, s/n, 50740-521 Recife, PE, Brazil; ^3^Departamento de Antibióticos, Universidade Federal de Pernambuco, Avenida Prof. Arthur de Sá, s/n, 50740-521 Recife, PE, Brazil

## Abstract

We describe the medicinal plants that have been reported to be antitumor agents and that have been used in ethnobotanic research in Brazil to answer the following questions: what is the abundance of plants reported to be antitumor in Brazil? Have the plant species used for tumor treatment in traditional Brazilian medicine been sufficiently examined scientifically? Our analysis included papers published between 1980 and 2008. A total of 84 medicinal plant species were reported to be used for cancer and tumor prevention or treatment; 69.05% of these were cited as being used for the treatment of tumors and cancer in general and 30.95% for specific tumors or cancers. The plants that were cited at a higher frequency were *Aloe vera*, *Euphorbia tirucalli*, and *Tabebuia impetiginosa*. At least, one pharmacological study was found for 35.71% of the species. Majority of the studies selected were conducted in rural communities and urban areas and in areas with traditional healers in Brazil. We found the following molecules to be the most studied in vitro and in vivo: silibinin, *β*-lapachone, plumbagin and capsaicin. The species addressed here constitute interesting objects for future studies to various professionals in the field of natural products.

## 1. Introduction

In Brazil, it is estimated that there will be more than 489,270 new cases of cancer in 2011 [1]. Also known as neoplastic tumors, malignant tumors are characterized by uncontrolled growth of transformed cells [2], which can rupture the basal membrane, attack and invade the surrounding tissues, and may metastasize [3]. 

There are some limitations in the treatment of cancer with chemotherapy, that in general provoke various toxic reactions [4]. In addition, solid tumors are generally resistant to chemotherapy due to the inability of the drugs to access hypoxic cells [5]. Moreover, many antineoplastic agents are not specific to cancer cells and can also damage healthy cells, especially those with rapid turnover, such as gastrointestinal and immune cells [2]. Because of this, many patients with cancer around the world have resorted to complementary and alternative therapies as adjuvant treatment in relation to official (radiation, chemotherapy, and surgery), as the use of plants [6]. 

Plant diversity is an important source of new molecules. More than 60% of the anticancer agents used today are derived directly or indirectly from natural sources [7]. Higher plants have been one of the largest sources of new compounds with pharmacological activity. For example, the species *Catharanthus roseus* (L.) G. Don (Apocynaceae) produces several alkaloids, two of which, vincristine and vinblastine, have anticancer activity [4]. *Taxus brevifolia* Nutt., pacific yew, produces a diterpenic alkaloid known as taxol, which has been shown to act against advanced ovarian cancer [8]. *β*-lapachone and lapachol are extracted from the bark of *Tabebuia impetiginosa* (Mart. ex DC.) Standl, which is native to Brazil; lapachol is sold in Brazil by Pernambuco Pharmaceutical Laboratory (LAFEPE) and is used for treatment of various neoplasias.

Ethnobotanical approach is a strategy that has successfully identified new bioactive molecules from diverse plants. In this approach, the information obtained from traditional communities about the use of medicinal plants is combined with chemical/pharmacological studies performed in laboratories [9]. This strategy has been helpful in plant pharmacological research and has yielded better results than the random approach used in different experimental models [10, 11].

Brazil is the country with the highest plant diversity on the planet, with approximately 55,000 species of higher plants [12] distributed in several ecosystems: Atlantic forest, Amazon Forest, Cerrado, Caatinga, Pantanal, and Pampas. In addition, the country also has an enormous cultural diversity that is reflected in the different ways its natural resources are used [13]. In general, the use of medicinal plants in Brazil is strongly influenced by the cultural miscegenation, the introduction of exotic species by Africans and Europeans since the times of the colonization and the native indigenous people who make use of the local plant diversity. Such immense plant and cultural diversity has favored the diversification of a popular pharmacopoeia based on medicinal plants [14]. 

This study presents a review of the medicinal plants reported in ethnobotanical studies conducted in Brazil that have antitumor properties. Our results aimed to answer the following two questions: (1) what is the abundance of plants reported to be antitumor properties in Brazil? and (2) have the plant species used for tumor treatment in traditional Brazilian medicine been sufficiently examined scientifically?

## 2. Materials and Methods

### 2.1. Survey and Study Selection

Our survey of ethnobotanical studies was performed using five databases (SCIELO, SCIRUS, SCOPUS, BIOLOGICAL ABSTRACTS, and WEB OF SCIENCE) using the following four combinations of keywords: ethnobotany AND Brazil AND medicinal plants, ethnobotany AND Brazilian medicinal plants, ethnopharmacology AND Brazil AND medicinal plants, and ethnopharmacology AND Brazilian medicinal plants. Our analysis included papers published between 1980 and 2008. From the studies obtained from our search, we selected only those of an ethnobotanical nature performed in Brazil and that cited at least one plant with popular antitumor properties. All information regarding the plant and its use, such as popular name, species, plant part, therapeutic indication, community type, biome, and location, where the study was carried out, was taken directly from the selected reports. For our analysis, we considered all of the plants as those that were popularly recommended for the treatment of tumors and/or cancer in general or for the treatment of more specific cancers, such as leukemia, warts, or cancers of specific organs or human body parts.

 Some studies did not specify the culture of the population studied (e.g., indigenous, farmers, quilombola, or urban), and in those cases, we designated it as a “local population.” The Brazilian ecosystems were classified as Amazon, Cerrado (Brazilian savanna), Atlantic forest, Caatinga (tropical dry lands), Pantanal (tropical wetland), and Pampas (South America plain), as defined by the Brazilian Institute of Geography and Statistics (IBGE). In those studies, where the biome type was not provided, the correct biome was obtained from the IBGE, to supplement the data.

The frequency or the number of times a given plant species was cited in the different studies analyzed was recorded. Pharmacological studies on the classified plants was verified in the aforementioned databases using the following keyword combinations: species name AND tumor and species name AND cancer. For the species names, all of the scientific synonyms listed in the database of the Missouri Botanical Garden (http://www.tropicos.org/) were used. For the evaluation of the pharmacological studies available for each plant species, we considered both *in vitro* and *in vivo* studies related to cancer or tumors in humans and animals.

## 3. Results

### 3.1. Survey of Ethnobotanical Studies

Out of 293 studies found using the different keyword combinations, 39 were selected according to the inclusion criteria. Of these selected studies, 89.7% were published between 2000 and 2008. We did not find any studies published during the 1980s. Six bibliographic review papers contained the highest number of plant species cited as antitumor ($\overset{̅}{X}$ = 8.67). Reports considered reviews were those that used data from the published primary literature. The average number of plant species cited, without taking reviews into account, was 2.15. If we took into account all studies including reviews, the average number of species cited was 3.74. The similarity between the plant species cited in the reviews and the primary literature was 29.76%.

Majority of the studies were performed in rural communities (farm areas) and urban areas and in areas with traditional healers (>82%). A minority of the studies were performed with indigenous and Quilombola populations. We found that a higher number of plants were cited as being antitumor in the Caatinga (27.38%; 23 spp.), Cerrado (25.0%; 21 spp.) and Atlantic forest (22.6%; 19 spp.), ecosystems. Communities in the Amazon Forest and the Pampas were each represented in 5.61% of the studies, which corresponds to six species. The studies that did not specify the type of vegetation or the location where the study was carried out but that were influenced by two different types of ecosystems corresponded to 20.51% of the studies, representing a total of 41 plant species cited. There was a 14.29% similarity between the species found in two or more ecosystems.

### 3.2. Survey of Ethnobotanical Studies

A total of 84 medicinal plant species were reported in the ethnobotanical/ethnopharmacological literature as being used for the treatment or prevention of cancer and tumors, and these species were distributed among 42 families and 63 genera (Table 1). The more highly represented botanic families were: Euphorbiaceae (9 spp.), Fabaceae (7 spp.), Apocynaceae (6 spp.), Asteraceae, and Vitaceae (4 spp. each). The genera with the highest number of species were: *Cissus* (4 spp.), *Himatanthus, Maytenus, Cnidoscolus, Euphorbia, Copaifera, Aloe*, and *Plantago* (3 spp. each). The plants most frequently cited were *Aloe vera* (eight)*, Aloe arborescens, Euphorbia tirucalli* and *Tabebuia impetiginosa* (each cited six times), and *Symphytum officinale* (five). Of these, only *Tabebuia impetiginosa* is native to Brazil.

Majority of the plant species were reported to be used for the treatment of tumors and cancer in general (69.05%), and a smaller proportion (30.95%) were reported to be used for the treatment of specific tumors, such as warts, leukemia, myoma, or cancers of the mouth, skin, and prostate. *S. officinale* stands out from the other plants because it was reported to be used for the treatment of cancer in general, leukemia and mouth and skin cancers. *In vitro* and *in vivo* studies related directly to tumors of animal origin were not found for 55 (64.29%) of the species analyzed here.

### 3.3. Survey of Ethnobotanical Studies

**Figure 1:**
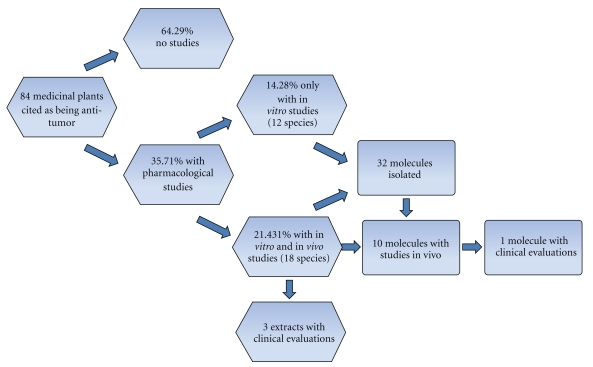
Level of pharmacological studies related to tumors of medicinal plants cited as being antitumor by traditional and nontraditional communities in Brazil.

**Figure 2:**
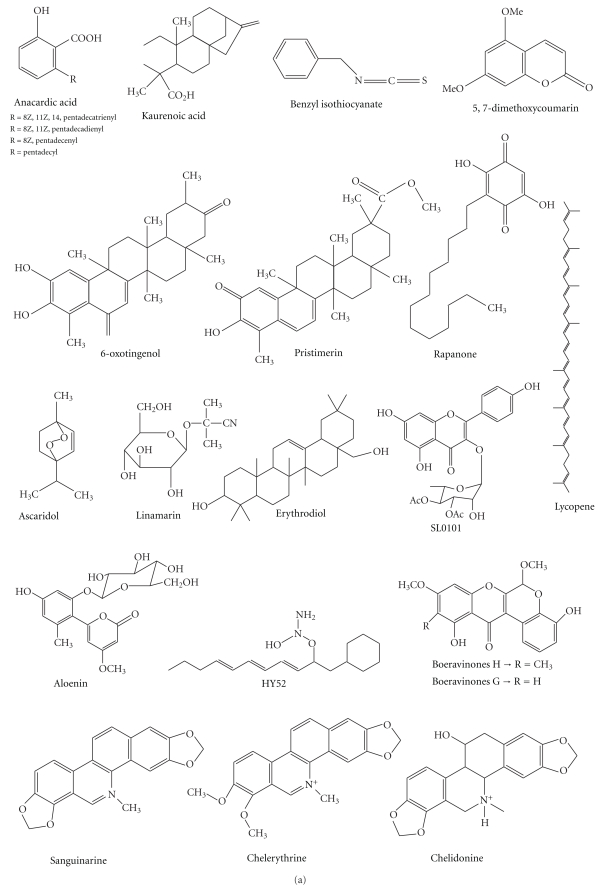

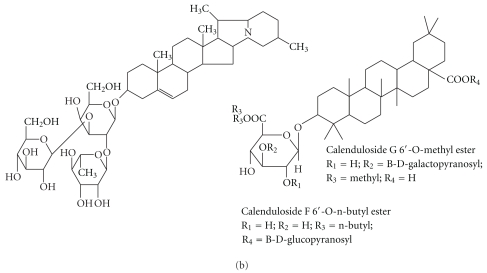
Molecules isolated from medicinal plants with antitumor activity *in vitro*.

**Figure 3:**
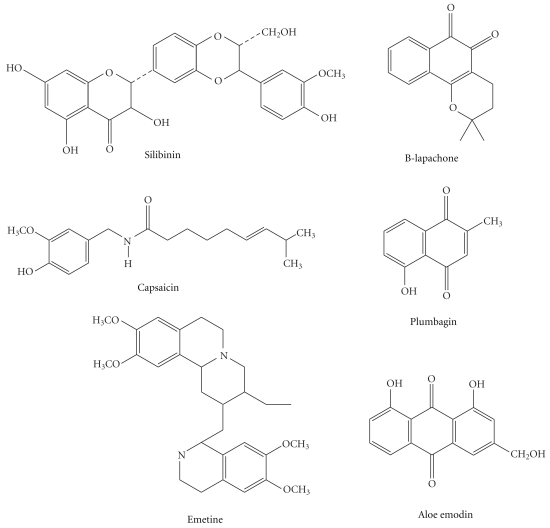
Molecules isolated from medicinal plants with antitumor activity *in vivo*.

We found at least one type of pharmacological study for 35.71% of the plant species (30 taxa). Considering only the plant species for which we found pharmacological studies, we found only 14.29% (4 taxa) of the species or their molecules were used for clinical studies, and 39.39% of the plants (12 taxa) were used to perform *in vitro* studies of their extracts or associated molecules (Figure 1). From the plants for which we found studies, approximately 30 molecules with *in vitro* and/or *in vivo* antitumor activity have been isolated (Figures 2 and 3).

Molecules or extracts that exhibited antitumor activity mainly act by inducing cell cycle arrest and/or apoptosis. Preclinical studies in cancer research have focused on the search for molecules that exhibit proapoptotic activity and promote cell cycle arrest. In our analysis, we found a great diversity of molecules of different chemical classes that exhibited anticancer activity, including alkaloids, peptides, glycoproteins, carotenoids, terpenes, carbohydrates, quinines, and phenolic compounds.

We summarize, in Table 1, the studies on the antitumor activity of each of the plant species we found in our search and the molecules that have been isolated from them.

## 4. Discussion

### 4.1. Survey of Ethnobotanical Studies

The highest numbers of studies citing antitumor plants were published between 2000 and 2008. This pattern is likely associated with the increase of publications relating to ethnobotanical studies conducted in Brazil during this time [106]. The low numbers of citations of plants identified as antitumor, with an overall average below 3 species per article, depicts in general, that local communities have a small repertoire of plants for the treatment of tumors when compared to more recurrent diseases and disorders, such as inflammation, flu, infections and parasites, which is focused on the most assiduous diseases. In a survey of the medicinal plants known and used by the people of the Caatinga biome, Albuquerque et al. [13] documented a total of 389 species, of which more than half were used to treat diseases of the digestive, respiratory, and genitourinary systems, while only 8 species (2%) were used to treat tumors. However, it is difficult to diagnose cancer using traditional medicine because the signs and symptoms of different types of cancer are not specific and are confused with those of other diseases.

Most of the selected studies were conducted in urban and farming areas and areas with traditional healers, probably because: (a) these populations have a greater number of studies in Brazil (see [106]), (b) these populations have a greater knowledge of the plants used to treat tumors, and (c) these populations are more susceptible to the influences of other cultures and other external sources, such as the media (similar to what happened to the wide dissemination of Red Lapacho (*Tabebuia impetiginosa*, by magazine O'Cruzeiro published in 1967 reports of miraculous healing in cancer patients)) [107] and the public markets (defined as public spaces where many types of products are sold, including medicinal plants and their derivatives, and as a forum for the exchange of cultural information) [23]. The low similarity between the plant species from the populations located in the different ecosystems (14.29%) suggests that the populations have a greater knowledge of medicinal plants from the local flora for the treatment of tumors.

### 4.2. Survey of Ethnobotanical Studies

Our survey of the 84 species of plants used in Brazil for the treatment of tumors likely represents only a fraction of the plants used for this purpose because studies published in local or regional journals may not be indexed in national and/or international databases. Additionally, not all communities in Brazil have been sufficiently studied. 

The Euphorbiaceae, Fabaceae, Apocynaceae, and Asteraceae families, which had the largest number of species represented in this study, were also the most represented in an ethnopharmacological study aimed at selecting plants for use in experimental studies; one of the criteria for species selection was they present categories of use predefined, among those cited for the treatment of cancers, tumors, ulcers, sore mouth, and throat [4]. Four plant species should be highlighted for being highly cited in the studies and for their greater presence in the different ecosystems and regions of Brazil: *A. vera, A. arborescens, E. tirucalli*, and *T. impetiginosa*. These plants are widely publicized in Brazil by the virtual media as having anticancer properties. Furthermore, there are several products containing *A. vera* and *T. impetiginosa* that are explicitly marketed over the internet for the treatment of cancer; however, these claims lack scientific support.

This scenario, the low numbers of citations of plants identified as antitumor considering only one local community studied, has some implications if we want to use an ethnobotanical/ethnopharmacological approach for the search of new molecules with antineoplastic activity derived from the Brazilian flora. First, targeted ethnobotanical/ethnopharmacological studies in specific communities can easily result in no or few species being directly reported as antitumor. Should one want to perform a pharmacological study with less than 100 plants (triage) based on the popular knowledge of a single community, other selection criteria will be required besides a suggestion that a plant can be used to treat cancer. For the selection of species used in popular medicine, Santos and Elisabetsky [4] took into consideration not only the direct evidence that a plant was used for the treatment of cancer and tumors but also the signs and symptoms associated with certain cancers, which were related to the cell lines available for screening, and reports of the toxic effects common to chemotherapeutic agents (quantified by the order of importance). These selection criteria resulted in the identification of a greater number of candidate species for use in laboratory studies.

Second, a greater number of ethnobotanical/ethnopharmacological studies conducted and data collected on a regional scale will increase the possibilities of identifying new molecules with antitumor effects. For example, using lung and breast cancer cell lines, Lee and Houghton [108] evaluated the *in vitro* cytotoxic activity of seven plant species used in traditional Malaysian and Thai medicine to treat cancer. Eleven extracts from six plant species (85.71% of the selected plants) exhibited antiproliferative activity against one of these cell lines, with IC_50_ values ranging from 2.7 to 35.8 *μ*g/mL. These extracts are of interest for future investigations.

### 4.3. Survey of Ethnobotanical Studies

The large number of plant species have not been analyzed for their antitumor potential (64.29%, 54 taxa) or have only been studied *in vitro* (14.29%, 12 taxa) indicates there is ample space in the field for future investigations of the anticancer activity of such plants in Brazil. In general, the plants that were used in various experimental studies showed significant results in the pharmacological models used, and these results corroborated their popular use. Many researchers selected plants used in alternative and complementary medicine to treat cancer, with the goal of evaluating the antitumoral activity. Satisfactory results has been found for both *in vitro* and *in vivo* [109, 110] activities, demonstrating that plant species used in popular medicine are a promising source for new molecules. 

We propose the following suggestions for the plant species surveyed in this study: (1) for plants that have not been analyzed for their antitumor potential by any pharmacodynamic study, it is necessary to perform *in vitro* experiments with different solvents and using different cancer cell lines;,(2) for species from which active extracts have been isolated, including *H. obovatus, A. hispidum, P. major*, and *S. occidentalis*, it is necessary to identify the molecule(s) responsible for their biological activity, (3) for the species from which active extracts with known chemical structure(s) have been isolated and which have demonstrated significant activity *in vitro,* it is necessary to proceed with studies *in vivo* (e.g., *V. odorata* (cicloviolacina), *C. officinalis* (calenduloside), *M. ilicifolia* (pristimerina, 6-oxotingenol and erythrodiol), *B. forficata* (HY52), and *C. langsdorffii* (kaurenoic acid)), and (4) for the species that have met the previous requirements, it is necessary to make an extensive *in vivo* biological evaluation and subsequently proceed with clinical evaluations (e.g., *S. marianum* (silibinin)*, T. impetiginosa* (*β*-lapachone)*, P. scandens* (plumbagin), and *C. frutescens* (capsaicin)). The species addressed here constitute interesting objects for future studies for the various professionals in the field of natural products.

## Figures and Tables

**Table 1:** Species of medicinal plants cited as being antitumor by traditional and nontraditional communities in Brazil with their plant parts used, indication, occurrence, and pharmacological studies.

Family/species	Plant parts used	Indication	Pharmacological study/molecules evaluated	Occurrence (reference)
Amaranthaceae				
*Iresine herbstii* Hook	Leaf	Cancer	—	2 [15, 16]
Anacardiaceae				
*Anacardium occidentale* L.	Resin	Wart	*In vitro*, *in vivo* and clinical/Anacardic acid, polysaccharides, oligosaccharides, *β*-galactose, and proteins [17–20]	2 [21, 22]
*Myracrodruon urundeuva* Allemão	Bark	Tumors, neoplasias	—	2 [23, 24]
Annonaceae				
*Rollinia leptopetala* R.E. Fr.	Bark	Tumors	—	2 [22, 25]
*Rolliniopsis leptopetala* (R.E. Fr.) Saff.	Bark	Tumors	—	1 [13]
Apocynaceae				
*Forsteronia refracta* Müll. Arg.	Latex	Cancer	*In vitro*/SL0101 (a kaempferol glycoside) [26]	1 [27]
*Hancornia speciosa* Gomes	Latex	Cancer	*In vitro* [28]	1 [29]
*Himatanthus articulatus* (Vahl) Woodson	Latex	Tumors, cancer	—	1 [22]
*Himatanthus bracteatus* (A. DC.) Woodson	Latex	Tumors, cancer	—	1 [22]
*Himatanthus obovatus* (Müll. Arg.) Woodson	Latex	Cancer	*In vitro* [30]	1 [31]
*Macrosiphonia velame* (A. St.-Hil.) Müll. Arg.	Whole plant, root	Tumors	—	1 [31]
Arecaceae				
*Orbignya phalerata* Mart.	Fruit	Leukemia	*In vitro* [32]	1 [22]
Asclepiadaceae				
*Marsdenia altissima* (Jacq.) Dugand	Bark	Cancer	—	1 [22]
Asteraceae				
*Acanthospermum hispidum* DC.	Leaf, flower, root	Cancer	*In vitro* and *in vivo* [33, 34]	2 [35, 36]
*Aster squamatus* (Spreng.) Hieron.	Aerial parts	Cancer	—	2 [15, 37]
*Calendula officinalis* L.	Whole plant	Cancer	*In vitro* and *in vivo*/calenduloside F 6′-*O-n*-butyl-ester and calenduloside G 6′-*O*-methyl ester [38]	1 [15]
*Silybum marianum* (L.) Gaertn.	—	Internal tumors	*In vitro* and *in vivo*/silybinin and silimarin [39, 40]	1 [16]
Bignoniaceae				
*Tabebuia impetiginosa* (Mart. ex DC.) Standl.	Bark, flower, bast	Cancer and tumors	*In vitro* and *in vivo*/*β*-lapachone [41, 42]	6 [13, 22, 43–46]
*Tecoma violacea *	Bark	Cancer	—	1 [47]
Boraginaceae				
*Symphytum officinale* L.	Leaf	Leukemia, cancer, mouth cancer, skin cancer	*In vitro* [48]	5 [23, 45, 46, 49, 50]
Caricaceae				
*Carica papaya* L.	Flower, fruit, latex	Wart	*In vitro*/5,7-dimethoxycoumarin, Lycopene and Benzyl isothiocyanate [51–53]	2 [13, 54]
Caryocaraceae				
*Caryocar coriaceum* Wittm.	—	Tumors	—	1 [55]
Cecropiaceae				
*Cecropia hololeuca* Miq.	—	Cancerous wounds	—	1 [56]
*Cecropia peltata* L.	—	Cancerous wounds	—	1 [56]
Celastraceae				
*Maytenus ilicifolia* (Schrad.) Planch.	Leaf, root	Cancer, skin cancer, tumors	*In vitro*/Pristimerin, 6-oxotingenol and Erythrodiol [57–59]	4 [16, 37, 60, 61]
*Maytenus obtusifolia* Mart.	Leaf	Cancer	—	1 [22]
*Maytenus rigida* Mart.	Bark	Cancer	—	1 [22]
Chenopodiaceae				
*Chenopodium ambrosioides* L.	Stem, leaf, whole plant	Cancer	*In vitro* and *in vivo*/ascaridol [62, 63]	2 [13, 31]
Cochlospermaceae				
*Cochlospermum regium* (Schrank) Pilg.	Root	Cancer	—	1 [31]
Crassulaceae				
*Cotyledon orbiculata* L.	—	Cancer	—	1 [64]
Euphorbiaceae				
*Cnidoscolus obtusifolius* Pohl ex Baill.	Leaf, flower	Cancer and tumors	—	3 [13, 35, 36]
*Cnidoscolus phyllacanthus* (Müll. Arg.) Pax & L. Hoffm.	Stem, bark, bast, látex, root	Wart	—	2 [13, 21]
*Cnidoscolus urens* (L.) Arthur	Root	Cancer	—	1 [23]
*Croton antisyphiliticus* Mart.	Leaf	Tumors	—	1 [65]
*Croton urucurana* Baill.	—	Cancer	—	1 [55]
*Euphorbia phosphorea* Mart.	Stem, latex	Wart	—	3 [13, 22, 47]
*Euphorbia prostrata* Aiton	Latex	Wart	—	3 [13, 22, 47]
*Euphorbia tirucalli* L.	Latex, aerial parts, leaf	Cancer and wart	—	6 [15, 16, 22, 64, 66, 67]
*Manihot esculenta* Crantz	Leaf, látex, root	Wart	*In vitro*/linamarin, esculentoic acids A and B [68, 69]	2 [13, 47]
Fabaceae				
*Anadenanthera colubrina* (Vell.) Brenan	Stem, bark, bast, flower, leaf, fruit	Cancer	*In vivo*/acidic heteropolysaccharide [70]	1 [13]
*Bauhinia forficata* Link	Leaf	Cancer	*In vitro*/HY52 [71]	1 [60]
*Copaifera langsdorffii* Desf.	—	Tumors	*In vitro*/kaurenoic acid [72]	1 [55]
*Copaifera multijuga* Hayne	Oil of fruit	Cancer	*In vitro* and *in vivo* [73]	1 [22]
*Copaifera reticulata* Ducke	Whole plant	Cancer	—	1 [22]
*Parapiptadenia rigida* (Benth.) Brenan	—	Tumors	—	1 [50]
*Senna occidentalis* (L.) Link	Leaf, seed, root	Cancer	*In vitro* [74]	2 [13, 36]
Iridaceae				
*Eleutherine bulbosa* (Mill.) Urb.	Leaf, bulb	Cancer	—	1 [67]
Lamiaceae				
*Leucas martinicensis* (Jacq.) R. Br.	Leaf	Benign tumors	—	1 [75]
Lecythidaceae				
*Cariniana rubra* Gardner ex Miers	Bark	Tumors (myoma)	—	1 [61]
Liliaceae				
*Aloe arborescens* Mill.	Leaf	Cancer, prostate cancer	*In vitro*, *in vivo* and clinical/Aloin [76–78]	6 [15, 16, 64, 66, 79, 80]
*Aloe soccotrina* DC.	Leaf	Leukemia	—	2 [22, 81]
*Aloe vera* (L.) Burm. f.	Leaf, root, stem and sap	Cancer	*In vitro* and *in vivo*/aloe-emodin and aloctin I [82, 83]	8 [13, 16, 23, 31, 55, 60, 84, 85]
Lythraceae				
*Lafoensia pacari* A. St.-Hil.	Bark	Cancer	—	1 [61]
Malvaceae				
*Abutilon grandifolium* (Willd.) Sweet	Leaf	Cancer and myoma	—	2 [16, 79]
Myrcinaceae				
*Rapanea guianensis* Aubl.	Branches with leaf	Tumors	*In vitro*/rapanone [86]	1 [87]
*Rapanea umbellata* (Mart.) Mez	Branches with leaf	Tumors	—	1 [87]
Myrtaceae				
*Myrciaria herbacea* O. Berg	Root	Cancer	—	1 [31]
Nyctaginaceae				
*Boerhavia diffusa* L.	Leaf, root	Leukemia	*In vivo*/punarnavine, boeravinones G and H [88, 89]	1 [31]
*Guapira pernambucensis* (Casar.) Lundell	Bark	Wart	—	1 [22]
Papaveraceae				
*Chelidonium majus* L.	Latex	Wart	*In vitro* and *in vivo*/chelidonine, sanguinarine, chelerythrine, and nucleases (CMN1 and CMN2) [90–92]	1 [15]
Piperaceae				
*Ottonia leptostachya* Kunth	Whole plant	Wart	—	1 [22]
*Piper regnellii* (Miq.) C. DC.	Leaf, aerial parts	Myoma	—	1 [37]
Plantaginaceae				
*Plantago australis* Lam	Leaf, root, whole plant, and inflorescence	Tumors, cancer and prevent cancer	—	3 [37, 50, 79]
*Plantago major* L.	Leaf, root, whole plant and inflorescence	Prevent cancer	*In vitro* and *in vivo* [93, 94]	1 [79]
*Plantago tomentosa* Lam.	—	Cancer	—	2 [16, 37]
Plumbaginaceae				
*Plumbago scandens* L.	Bark, leaf, root, whole plant	Wart	*In vivo* and *in vitro*/plumbagin [95, 96]	4 [13, 21, 22, 25]
Pteridaceae				
*Adiantum raddianum* C. Presl	Aerial parts	Cancer	—	2 [37, 64]
Rubiaceae				
*Psychotria ipecacuanha* (Brot.) Stokes	Whole plant	Cancer	*In vitro* and *in vivo*/emetine [97, 98]	1 [23]
Sapindaceae				
*Cardiospermum oliveirae* Ferrucci	Stem, leaf, flower, aerial parts	Tumors	—	3 [13, 22, 36]
*Serjania erecta* Radlk.	Leaf, root	Cancer	—	1 [61]
Simaroubaceae				
*Simaba suffruticosa* Engl.	Root	Cancer	—	1 [31]
Solanaceae				
*Capsicum frutescens* L.	Leaf	Tumor	*In vitro* and *in vivo*/capsaicin [99, 100]	1 [60]
*Solanum americanum* Mill.	Leaf	Myoma	*In vitro* and *in vivo*/glycoprotein and solanine [101, 102]	1 [60]
*Solanum paniculatum* L.	Root, leaf, fruit	Internal tumors	—	1 [87]
Tiliaceae				
*Luehea paniculata* Mart.	Bark, leaf	Tumors	—	1 [103]
Turneraceae				
*Turnera ulmifolia* L.	Leaf, root, whole plant	Cancer	—	1 [13]
Verbenaceae				
*Stachytarpheta cayennensis* (Rich.) Vahl	Leaf	Cancer	—	2 [37, 64]
*Vitex triflora* Vahl	Leaf, latex	Wart	—	2 [22, 104]
Violaceae				
*Viola odorata* L.	—	Cancer	*In vitro* and *in vivo*/cycloviolacin O2 [105]	1 [64]
Vitaceae				
*Cissus coccinea* (Baker) Mart. ex Planch.	Leaf, root	Wart	—	1 [22]
*Cissus decidua* Lombardi	Stem, leaf, flower, aerial parts	Cancer	—	3 [13, 22, 36]
*Cissus duarteana* Cambess.	Sap and root	Wart	—	1 [103]
*Cissus erosa* Rich.	Aerial parts	Wart	—	2 [21, 22]
Zingiberaceae				
*Costus spiralis* (Jacq.) Roscoe	—	Prostate cancer	—	1 [84]
